# Engaging national organizations for knowledge translation: Comparative case studies in knowledge value mapping

**DOI:** 10.1186/1748-5908-6-106

**Published:** 2011-09-12

**Authors:** Joseph P Lane, Juan D Rogers

**Affiliations:** 1 Center on Knowledge Translation for Technology Transfer, University at Buffalo (SUNY), Amherst, NY, USA; 2 Georgia Tech University, School of Public Policy, Atlanta, GA, USA

## Abstract

**Background:**

Government sponsors of research and development, along with their funded investigators, are increasingly tasked with demonstrating evidence of knowledge use by nontraditional audiences. This requires efforts to translate their findings for effective communication. For technology-related knowledge, these audiences include clinicians, consumers, manufacturers, public policy agencies, and knowledge brokers. One potentially efficient approach is to communicate research findings through relevant national organizations. However, this requires an understanding of how such organizations view and treat research knowledge, which can be determined through knowledge-value mapping. Do knowledge values differ between national organizations representing different audiences? Can a deeper understanding of knowledge values help sponsors, investigators, and organizations better communicate research findings to stakeholders?

**Methods:**

A series of comparative case studies on knowledge-value mapping were derived through interviews with spokespersons for six national organizations. The semi-structured interviews followed a 10-item questionnaire to characterize different ways in which each organization engages with research-based knowledge. Each participating organization represents a particular stakeholder group, while all share a common interest in the research subject matter.

**Results:**

Each national organization considers the value of the research knowledge in the context of their organization's mission and the interests of their members. All are interested in collaborating with researchers to share relevant findings, while they vary along the following dimensions of knowledge engagement: create, identify, translate, adapt, communicate, use, promote, absorptive capacity, and recommendations for facilitation.

**Conclusions:**

The principles of knowledge translation suggest that investigators can increase use by tailoring the format and context of their findings to the absorptive capacity of nonscholars. Greater absorption should result in higher levels of knowledge awareness, interest, and use, which can then be documented. National organizations and their members, in turn, can strive to optimize their absorptive capacities regarding the state of the sciences. This combination will ensure the highest possible return on public investment in research activities. This knowledge-value mapping study concludes that national organizations are appropriate channels for communicating research findings and for meeting statutory requirements and general expectations for generating and documenting knowledge use.

## Background

### Research value to society

Government agencies around the globe sponsor research, either internally through government laboratories or externally through universities and affiliated organizations. Over the past decade, these sponsoring agencies and their programs have come under increasing scrutiny to demonstrate evidence showing how outputs from research result in beneficial impacts for society. In the United States, this scrutiny is grounded in prior law through the Government Performance Results Act enacted in 1993, which holds government programs accountable for achieving intended results, including sponsored research programs [1]. Similarly, the European Commission has increased the importance of considering societal impacts within their Framework Programmes, including determining how to define and measure such impacts.

Increasing expectations for accountability presents a new challenge for all involved. In order for sponsor agencies and grantees to demonstrate evidence that research findings have utility to stakeholders outside of the academic system, they need to identify and reach these nontraditional targeted audiences. Of course, no single investigator can be expected to communicate directly with exponentially larger and more diverse audiences. This paper explores one option to meet this expectation: to identify and exploit existing channels for networked communication, through national organizations operating in the field of interest. Furthermore, since the process of use of knowledge by nonacademics is a complex process of social communication, the paper suggests a means for obtaining a better understanding of what factors may facilitate or hinder the use of research results by each stakeholder group [2].

### Knowledge translation as a broad communication strategy

Knowledge translation (KT) has emerged as a communication strategy to increase relevance and use of completed research discoveries in health-related fields and to increase the societal relevance of ongoing research [3]. Many specific translation strategies depend on the content of the substantive research results and the contexts in which they are expected to be applied. Therefore, structured approaches, such as the Knowledge to Action (KTA) Model promulgated by the Canadian Institutes for Health Research [4], have emerged for improving communication about research findings to various target audiences. The KTA Model instructs the researcher on how to consider and incorporate the context of any potential user audience into their plans for translating knowledge into action [5].

However, it is important to recognize that government-funded projects are not limited to scholarly research activity. Some government programs also sponsor technology-based projects that go beyond research, to include development activities where the research-based concepts are reduced to some practical form, such as a prototype invention. Still other government programs extend the project's mission to conducting production activities, where the development outputs become finished devices or service innovations for the marketplace. Each of these methods are somewhat codified in their respective literature and practice standards, having their own levels of rigor and relevance appropriate to their state of knowledge [6]. Such technology or product-oriented programs are designed to address a national need (*i.e*., military weapon systems) or to solve a societal problem (*i.e*., assistive technology for persons with disabilities), where public funding is justified to address issues not amenable to standard market forces.

One might then ask, once we integrate development and production methods with research methods into a broader process, can we still treat the successive outputs as knowledge for translation purposes? The authors' assert that KT remains an appropriate strategy because the novel kernel of knowledge from the original research remains as it transitions from the state of research discovery through the other two knowledge states of development invention and industry innovation. However, as the kernel of knowledge transitions from one state to another, it may be decoupled from the original investigator and sponsor, particularly if those actors are not actively involved in these downstream and possibly independent transitions.

This situation of translating technology-based knowledge illustrates what is at stake for KT in general. There is more than one collection of actors involved in the activities and behaviors spanning processes from knowledge creation to knowledge use. So there is interest in tracing the original scientific (research) contribution to latter states of knowledge, as well as in understanding the variables influencing awareness, interest, and use of research-based knowledge in downstream activities. Of paramount importance to all is for the kernel of knowledge to progress through the chain of stakeholders and the sequence of methods, with the highest probability of success. For technology-based knowledge, success is defined as beneficial socioeconomic impacts.

### Knowledge-value mapping as a knowledge-translation tool

Given the multiple knowledge states and multiple relevant stakeholder audiences described above, active involvement in KT may be the only way for researchers and their sponsors to maintain a trail of evidence from their findings to the eventual applications. Projects and investigators lacking this commitment to active engagement are less able to demonstrate evidence of impacts across multiple stakeholders and over time.

KT strategies require the knowledge creator--or possibly some intermediaries--to convey the research findings in a form with appropriate content perceived form perceived as useful by the target audience. Tailoring the message to the recipient is expected to increase the likelihood that the knowledge will be understood (comprehension) and then implemented in some practical form (behavior). To this end, a team from the Georgia Institute of Technology described "knowledge-value mapping" as an approach to exploring the values held by target audiences toward research, so that a message about new research findings can be tailored to connect with those values [7]. The authors of this paper contend that the value of knowledge is only realized when it is applied. Once implemented by individuals within one or more stakeholder groups, the knowledge demonstrates value by generating artifacts in the form of outcomes and impacts. Knowledge-value mapping (KVM) allows knowledge creators or their intermediaries to construct a map of potential knowledge flows and to identify factors either facilitating or hindering the use of knowledge [2,8].

The KVM concept appears appropriate for application to knowledge outputs in any of the three states of discovery, invention, or innovation. Various stakeholder groups may differentially value knowledge in various states. Researchers traditionally prepare publications for other scholars. They are now tasked with considering what other audiences might benefit from their findings and how each audience might respond to the knowledge in its current state. For many research projects, and certainly for development projects generating technology-based inventions, these audiences necessarily also include manufacturers, clinicians, consumers, policy makers, and brokers. All of these other audiences participate in the process of moving discoveries and inventions to the marketplace in the form of innovations. The diversity of audiences and the likely diversity of their value systems raise a host of questions. How can one efficiently reach a wide range of audiences, each with different value systems regarding the awareness, interest, and use of new knowledge from research? What other factors besides understanding the content of the knowledge may be at stake to encourage its use? For example, a growing body of literature demonstrates that if new approaches to clinical treatment involve changing the role of health workers, many barriers to implementation arise based on values and procedures beyond the actual medical efficacy of the new approach [8].

It is not always feasible to communicate research-based knowledge directly to potential users on a one-to-one basis. There may be multiple mediations of the knowledge that originated in research before it reaches potential users. There may be one or more tiers of intermediary organizations that can serve as a surrogate for effectively communicating knowledge within the context and values of the target audience, for example, national organizations that represent a profession that depends on an area of scientific knowledge (*e.g*., physicians, clinicians, engineers) or potential knowledge beneficiaries (*e.g*., employers or recipients of products or services). National organizations understand and likely share the values of their constituencies, which they can represent to the knowledge creator. Could these national organizations serve as a conduit for efficiently and effectively communicating new knowledge to their members? Will their credibility make members more inclined to pay attention to materials received?

Rogers & Martin [9] applied KT principles to a specific issue involving a federal lawsuit by a national organization representing persons with visual impairments, which claimed that the U.S. Department of the Treasury was not in compliance with current laws requiring accessible currency. The interesting point is that although the science and technology underlying a solution were understood, the knowledge application was blocked by the competing values of several stakeholder groups holding opposing views.

Rogers & Martin classified members of these groups in terms of their relevant knowledge, relevant values, and role in the use of knowledge concerning the issue of accessible currency. The KVM exercise identified opportunities for enabling KT to occur within and between the opposing sides of the case.

The current study explores how national organizations can play a crucial role in communicating new knowledge to diverse audiences, how their organization's context shapes their values regarding research-based knowledge, and how creating a detailed map of their respective values can help plan a KT strategy.

### Study of national organizations involved with augmentative and alternative communication assistive technologies

This KVM exercise involved the field of assistive technology devices and services, and more specifically focused on assistive technology for persons lacking the ability to communicate verbally. This is called augmentative and alternative communication (AAC). The study focuses on the knowledge values of national organizations with members who have an interest in the identification, communication, and application of research-based findings within AAC.

This KVM exercise was conducted as part of a broader ongoing study examining the effectiveness of three different approaches to communicating new research-based knowledge: (1) traditional passive diffusion, (2) targeted knowledge dissemination, and (3) tailored/targeted KT. The broader study involves a randomized controlled trial to compare stakeholder awareness, interest, and use of new AAC knowledge before and after various experimental interventions. The aim here is to consider how KVM of national organizations can help knowledge creators identify opportunities for communicating their research findings more efficiently and effectively than attempting to contact members of diverse stakeholder groups individually.

This analysis involved three research questions:

1) Are national organizations appropriate conduits for communicating research-based information to entire groups of individuals?

2) What are the value systems of these national organizations regarding research-based knowledge, as we may articulate them with information gleaned from a semi-structured interview process?

3) What guidance on how best to communicate research-based knowledge to these organizations, and through them to their members, can we obtain from mapping the knowledge values of national organizations?

## Methods

### Multiple comparative case studies

The project team previously identified six generic categories of key stakeholder groups likely to have an interest in using technology-oriented research and development outputs [10]. Based on those generic categories, we brought our team's own knowledge of AAC stakeholders to consultations with experts in the field of AAC, where we identified more specific categories of persons considered to be appropriate target audiences for the AAC output under study. These categories were as follows:

1. Manufacturers of AAC devices that might integrate the knowledge in products

2. Clinicians specializing in AAC who might recommend the knowledge to clients

3. Consumers who are adult AAC users and might apply the knowledge directly

4. Researchers who might be investigating related AAC issues

5. Brokers in a position to refer clinicians or adult consumers to the knowledge

6. Policy makers (or policy implementers) concerned with AAC issues

The project team continued to work with AAC experts to next identify specific national organizations representing one or more of these target audiences, with at least a portion of members likely interested in new knowledge regarding adults (persons over 18 years old) who use AAC devices. Through an intensive review process, we identified five organizations deemed appropriate. A sixth organization--which happens to also represent members of the five other stakeholder groups--participated in a pilot test of the data collection instrument.

The national organizations representing the target audiences are as follows:

1. Manufacturer stakeholders: Assistive Technology Industry Association (ATIA), http://www.atia.org/i4a/pages/index.cfm?pageid=1

2. Clinician stakeholders: American Speech-Language Hearing Association (ASHA), http://www.asha.org/

3. Consumer and researcher stakeholders: International Society for Augmentative and Alternative Communication (ISAAC), http://www.isaac-online.org/en/home.shtml

4. Broker stakeholders: Association on Higher Education and Disability (AHEAD), http://www.ahead.org/

5. Public policy stakeholders: Office of Special Education and Rehabilitative Services (OSERS), http://www2.ed.gov/about/offices/list/osers/index.html

6. Cross-stakeholder organization (pilot study): Rehabilitation Engineering & Assistive Technology Society of North America (RESNA), http://www.resna.org

For this study, each national organization constituted a case for a multiple comparative case study design [11]. We attempted to identify the core values of each organization that affect the flow of research results to potential beneficiaries in their constituencies. For this purpose, we conducted semi-structured interviews to understand how these organizations identify and apply research-based knowledge in order to determine the priorities that characterize their role in the flow of knowledge toward the context of use. The interview protocol is shown in Additional file 1, appendix A.

The design addresses 10 major areas in which the priorities of the organizations may affect their involvement with research knowledge and its communication and use. The first six sequentially explore ways each organization interacts with knowledge drawn from research activity. These are

1. creating knowledge: conducting research internally or funding others to conduct research for the organization;

2. identifying knowledge: searching for research findings that have already been generated by others;

3. translating knowledge: paraphrasing research findings to make them more relevant or understandable to the target audience;

4. adapting knowledge: interpreting research findings to improve their fit within the organization's context;

5. communicating knowledge: disseminating or demonstrating research findings through various media channels;

6. using knowledge: applying research findings to situations within the organization or its body of members.

The next two areas address how the organization promotes the use of research knowledge among the membership or constituency. Another assesses the capacity of the staff/membership to understand, assess, and apply research-based knowledge. Finally, recommendations were sought from each organization for facilitating the communication of such knowledge to and through the organization.

### Case study process

For each national organization identified, the project team followed a chain of contacts to eventually reach the person responsible for identifying and communicating research-based information. In some cases this person was the organization's director or deputy director, and in others it was a division head responsible for research activity.

Once in contact, that person received a summary of our project and an explanation of this KVM exercise. We asked for their permission to engage in a telephone-based interview likely to require one to two hours. In exchange, we offered an honorarium to the organization--except for OSERS, which could not accept payment as a federal organization.

The project's interview protocol was previously submitted to the University at Buffalo's Institutional Review Board (IRB), to determine the level of human-subject protection or informed consent required. The IRB required verbal consent for participation, first from the professional organization's management, then from the individual identified as the spokesperson. In each instance, they were briefed on the study and given an advance copy of the interview questionnaire to review. Each interview commenced after verbal consent was obtained.

The interviews were conducted in a two-stage arrangement. First, the interviewee(s) reviewed the KVM questionnaire so they could either familiarize themselves or even complete the answers in advance. They were asked to return responses prior to the scheduled telephone interview. This permitted the project team to review the organization's initial responses and formulate probing follow-up questions during the interview. Second, they participated in a verbal interview via teleconference. Some cases required a follow-up interview to clarify responses, or to give the interviewee(s) additional time to respond to the open-ended questions.

Based on the in-depth telephone interviews, the project team expanded or revised the responses previously sent in by the organization representative and transcribed the responses into a spreadsheet to permit comparisons. The team also added notes where appropriate to document follow-up questions or clarify responses in the context they were made. The resulting document became the basis for the following qualitative analysis.

## Case study results

### 1. Priorities related to creating research knowledge

The findings from research studies are a valued asset for all six organizations. All but one directly engaged in some kind of research activity at least occasionally. While not currently engaged in any research, ATIA recently formed a committee to explore how best to integrate research activity and findings into this industry association.

As a government entity, OSERS funds extramural research projects to improve quality of life for persons with disabilities, particularly to advance education, employment, rehabilitation, and independent-living outcomes, across all fields of application. ASHA conducts member surveys, maintains a national database of provider-reported information, conducts literature syntheses, and sponsors external research activities, all of which support the practitioners in the field and their students in training. As interdisciplinary organizations representing multiple stakeholder groups, ISAAC, RESNA, and AHEAD orchestrate research activity funded by and performed by others. This includes practice standards development, professional development, and policy formulation.

Five organizations publish peer-reviewed journals containing reports of applied research studies, with two of them (ATIA's and AHEAD's journals) freely available through open access:

• ISAAC: *Augmentative and Alternative Communication*

• ASHA: *Journal of Speech, Language and Hearing Research*

• ATIA: *Assistive Technology Outcomes and Benefits*

• RESNA: *Assistive Technology*

• AHEAD: *Journal of Postsecondary Education and Disability*

In sum, these organizations may be considered active intermediaries of the flow of research knowledge. They act as brokers and communicators of research results and have extensive networks to many potential users. They appear to be important actors in the KT process, be it systematic and intentional or spontaneous and informal.

#### Creators and users of internal research

AHEAD and ASHA have internal research staff, ISAAC and OSERS engage contractors or grantees, while RESNA involves both internal and external personnel to create new knowledge through research methods. Internal research staff should be identified as key points of contact for communicating external research knowledge relevant to the organization's mission. External researchers should track contract/grant opportunities in their areas of content expertise.

As shown in Table 1, each organization targets different combinations of knowledge users as their intended audiences. All organizations target clinicians/practitioners and educators/employers, which is expected given their shared interest in AAC technologies and users. All but ISAAC target public policy agencies as an audience. Four generate internal research findings for use by manufacturers/suppliers. Three target their internal staff, and three target consumers/family members. RESNA is the only professional association to report nonmembers as part of their target audience, including community-based organizations that may be able to apply research-based knowledge. As a government agency, by statute, OSERS targets a wide range of constituent groups, including school staff and administrators, parents, counselors, community-agency directors, and grantees of OSERS' three internal agencies: Rehabilitation Services Administration, National Institute on Disability and Rehabilitation Research, and Office of Special Education Programs.

**Table 1:** Target audiences for internally-generated research findings

	National organization					
**Audience**	**ATIA**	**AHEAD**	**ISAAC**	**ASHA**	**OSERS**	**RESNA**

Clinicians and practitioners	X	X	X	X	X	X

Consumers and families			X		X	X

Policy makers	X	X		X	X	X

Educators and employers	X	X	X	X	X	X

Manufacturers	X		X		X	X

Nonmembers	X				X	

ATIA = Assistive Technology Industry Association; AHEAD = Association on Higher Education and Disability; ISAAC = International Society for Augmentative and Alternative Communication; ASHA = American Speech-Language Hearing Association; OSERS = Office of Special Education and Rehabilitative Services; RESNA = Rehabilitation Engineering & Assistive Technology Society of North America.

These results show how diverse the patterns of knowledge flow to various stakeholders can be. Each national organization has formulated a different approach to managing stakeholder interactions given the different ways in which these constituents use research-based knowledge. These linkages are crucial in the process of KT. For example, a researcher seeking to communicate AAC findings to consumers or manufacturers will likely obtain the most collaboration from ISAAC and RESNA, while OSERS may be receptive to integrating these findings within their internal documentation or state-of-science summaries. Furthermore, this result suggests that further study of the interactions of these organizations with specific constituencies would be necessary in order to determine what the main challenges of KT are for potential uses of research results deemed beneficial to those constituencies.

These national organizations are already playing a KT role on behalf of their members. Such a role can be supported and expanded through collaborations with research sponsors and investigators who are committed to more efficient and effective communication with likely knowledge users.

### 2. Priorities related to identifying research knowledge

Two organizations (ASHA and OSERS) search for new research findings very frequently--one might say constantly. ISAAC and RESNA search frequently, while ATIA and AHEAD occasionally search for new research findings.

OSERS searches continuously for new findings to inform internal staff, support the content of grant/contract solicitations, update statutes and regulations, monitor grantee/contractor performance, and provide policy advice to other government agencies. Clearly, its close proximity with research activities is a key position to leverage new research findings in multiple ways at a high level of visibility and potential impact.

ASHA continuously searches for new findings in support of three programs: (1) informatics--requires updates on surveillance and epidemiological data, for assessing needs for, and impact of, AAC services and regulations; (2) education--keeping members informed about current AAC findings; and (3) dissemination--content for a column on current research findings in both print and e-zine publications for members.

ISAAC and RESNA both search frequently to keep their memberships informed about current findings and in support of their journals and newsletters. As interdisciplinary and cross-sector agencies, both ISAAC and RESNA communicate research findings to maintain relevance with their various constituents and to generate reference material within their core knowledge base. Both organizations use research knowledge strategically to inform public policy agencies. ATIA and AHEAD both search for new findings occasionally to support their journals and to maintain the dissemination of relevant findings to their members. As an industry-focused organization, ATIA seeks research information that companies can apply and is interested in brokering partnerships between researchers and companies that can apply their findings.

Monitoring new research findings and communicating them to members is an excellent way for organizations to demonstrate added value. The Assistive Technology Industry Association's efforts to broker partnerships between academia and industry add a new dimension to the critical but complicated relationship between the two sectors that typically operate independently. Their efforts are in line with the trend of emergent intermediations between universities and industry to facilitate collaboration and technology transfer. These have taken shape mostly on university campuses in the form of specialized contracting and intellectual property offices, extension services, and incubators, among other things [12]. The third-party brokerage represented by ATIA is a confirming instance of the potential for engagement between sectors with diverse value systems regarding research-based knowledge.

ISAAC and RESNA focus communication efforts on government agencies, as research-based findings are often viewed as more objective than the opinions of the organizations themselves. Knowing the purposes to which findings are applied helps external researchers identify those organizations most receptive to their findings and helps them tailor the message conveyed by the findings to the specific interests of the target national organization.

#### Sources of new research knowledge

All six national organizations search academic journals (both online and in print) and all but AHEAD search training programs and conference proceedings. The organizations were willing to name specific journals they monitored, which is helpful for researchers attempting to properly position their work.

However, formal publication is not a requirement for consideration. All six organizations also peruse websites on relevant topics to identify research-based knowledge, and four of the six search white papers and other internal reports from other sources. Ensuring that work is visible--that is searchable in electronic form--may be a critical aspect of positioning. Even if findings are published in a peer-reviewed journal, one may wish to create a keyword-laden summary for a website or post a white paper version as another opportunity to be found by search engines.

All the national organizations studied in this project seek input from individuals with expertise on particular research topics. As facilitators of the knowledge flow, these organizations engage directly with prominent members of the research community. They enable knowledge producers and users to increase awareness of each other's needs and priorities. In the process, they reduce the transaction and opportunity costs of these interactions. Researchers may gain substantial dissemination and translation benefits by becoming acknowledged as an expert in a particular topic area.

#### Assessment of quality of research findings

Most organizations search multiple sources for research findings, some of which lack quality controls such as peer review (*e.g*., white papers and websites). To what extent do organizations recognize the need for a standard of rigor and the means applied to screen findings prior to internal circulation? Their organization's standards also reveal the main priorities underlying their search for relevant research knowledge.

These organizations are all aware of the need for quality assurance--particularly since most publish peer-reviewed journals or operate juried conferences. To the extent they are referencing white papers or web postings, the organizations seek corroboration from other sources, such as companion publications under peer review or other works by the same author. They also rely on their identified external experts to help screen findings--another incentive for being viewed as an expert in a specific topic area. In sum, the main quality standards are taken from the research community itself. However, the priorities of usability of results are embedded in the topic selection that is prior to the assessment of quality of the results of their search.

Some of the organizations have charged committees with establishing review criteria to assess the quality of research conducted by others. One of the critical areas of concern is methodological rigor because that establishes the credibility of the findings. Some organizations also judge the quality of the writing. Poor presentation of materials reflects on the author and reduces the material's ability to communicate effectively to constituents.

Descriptions of high-quality research designs, along with explanations of the findings' relevance to various stakeholders, are key to creating interest in the findings and motivating the organizations to reference and disseminate either the full study or a synopsis. These national organizations seek reports simultaneously demonstrating both high rigor and high relevance.

### 3. Priorities related to translating research knowledge

The definition of KT used in the interviews included an option of "paraphrasing research findings to make them more relevant and understandable...." These national organizations were reluctant to paraphrase the research findings of others. Only two organizations reported doing so either very frequently (ASHA) or frequently (OSERS). OSERS staff distill materials from multiple sources for communication to other internal staff, to other government programs, or to incorporate the findings into statutes, regulations, and requests for external proposals. ASHA, as a professional and credentialing organization of clinicians, takes an active role in communicating research information in special formats that involve interpretation of the research results for the needs of their audience.

Respondents agree that the investigator's form and content of research findings should be preserved. Only organizations directly involved with research reported having the competencies to carry out such interpretations. Others avoided it out of concerns about altering the original meaning of the findings. They thought that any paraphrasing should be left to the potential user of the information. In cases where translation was necessary, they contacted the original author to either revise the material or to present their work to internal staff. ISAAC was the exception. As an organization closely associated with consumers, ISAAC was skeptical about the ability of researchers to effectively translate findings for the point of knowledge application, so they preferred to sponsor translation independently.

When organizations did resort to paraphrasing, they strove to maintain the integrity of the author's original study and findings. There is widespread deference to the author's original work, which is evidence that the author should exercise great care when preparing the original manuscript. It is reassuring for researchers to know that these national organizations will safeguard the author's work. Conversely, the same deference reinforces the author's obligation to lead efforts to translate or paraphrase the original manuscript to effectively communicate the findings to various stakeholder audiences.

### 4. Priorities related to adapting research knowledge

The organizations we studied fell essentially in two opposite camps on this matter. Three engaged in the adaptation of knowledge (albeit two did it only occasionally) while three did not. Here again the crucial issue was internal capabilities to link the research to specific needs of their constituencies. In open-ended conversations, several organizations expressed even greater reservations about adapting knowledge than about translating knowledge. They considered adaptation to be synonymous with modification, which they opposed due to the high potential to change the original author's meaning.

Both ISAAC and RESNA report occasionally adapting findings to foster dialogue between the physical science and social science disciplines within their membership. ISAAC reported that members may need to adapt knowledge to permit its absorption within their culture. This is a direct consequence of the diversity of applications and needs that the consumer community has. It is very difficult for one organization to have the capabilities to address all of them at the same level of expertise. RESNA's adaptation occurs in the preparation of position papers, standards/guidelines, quality indicators, and benchmarking, where consolidating and reconciling a wide range of findings is necessary. The knowledge adaptation is seen as a step beyond translation in those instances where further effort is necessary to make the knowledge understandable or relatable to their members' own context.

OSERS reports knowledge adaptation frequently in the context of distilling knowledge from multiple sources to address the agency's multiple missions. Further, the agency has to position its own knowledge into the context of its broader cabinet-level agency (U.S. Department of Education). OSERS adapts and applies research-based knowledge to demonstrate how government-sponsored projects, programs, and policies relate to persons with disabilities and their quality of life. OSERS must also adapt knowledge for strategic reasons, to represent the interests of their public constituencies within broader policy issues where those interests might not otherwise be considered.

The main theme is that KT must address a diversity of audiences for the contextualization of research findings to result in more effective communication.

### 5. Priorities related to communicating research knowledge

All six organizations reported being highly engaged in communicating research-based knowledge. All view their electronic media (email, listserv, websites) as prime vehicles for communicating research findings. All also reported conference proceedings, presentations, and workshops as equally popular approaches. Five organizations have their own peer-reviewed journals that constitute a direct mechanism for communicating research knowledge.

ATIA, ASHA, and OSERS all reported webcasts/webinars and special interest groups as frequently used methods of communicating research knowledge. ATIA and RESNA both use white papers or position papers frequently, possibly because both have significant memberships from industry and these are common approaches within that sector. ATIA was the only organization to report using popular media (*i.e*., television).

Due to its unique mission as a government agency, OSERS reports using small group meetings with policy makers and staff members in government agencies as a mechanism for communicating research findings about persons with disabilities that are relevant to broader statutory, regulatory, or programmatic issues.

Table 2 below shows the range of stakeholder groups considered to be target audiences for dissemination through each national organization. For example, all six organizations consider some elements of clinicians and practitioners to be target audiences. When asked about complications arising from the communication of research to others, several respondents mentioned the need to disassociate the organization from the research reported, by including disclaimers to avoid perceptions of endorsement. Some also reported concerns about the inability of the organization to control how the recipients will interpret the message or how they will apply any new knowledge communicated through the national organization. This is another challenge for KT because researchers cannot control how audiences apply, translate, adapt, or communicate the findings to others.

**Table 2:** Target audiences for dissemination through national organizations

	National organization					
**Audience**	**ATIA**	**AHEAD**	**ISAAC**	**ASHA**	**OSERS**	**RESNA**

Clinicians and practitioners	X	X	X	X	X	X

Consumers and families	X		X	X	X	X

Policy makers	X	X		X	X	X

Educators and employers	X	X	X	X	X	

Manufacturers	X		X		X	X

Others	X				X	

ATIA = Assistive Technology Industry Association; AHEAD = Association on Higher Education and Disability; ISAAC = International Society for Augmentative and Alternative Communication; ASHA = American Speech-Language Hearing Association; OSERS = Office of Special Education and Rehabilitative Services; RESNA = Rehabilitation Engineering & Assistive Technology Society of North America.

### 6. Priorities related to using research knowledge

The KVM questionnaire also explored various ways in which research-based knowledge could be used and solicited examples of knowledge use.

Five organizations reported internal use of research-based knowledge. All five referenced academic journals, while three also referenced websites, training seminars, and conferences. Three also reported using findings from internal projects or commissioned/sponsored external activity. ATIA responded that the question was not applicable to them as an industry organization because their use of knowledge is not focused internally but only externally to their constituents.

Since KT is in essence a social communication problem [2], the use of multiple media for publication of research results is critical. Therefore, academic journals are not the only source of research-based findings. The other sources cited represent opportunities for scholars to increase the likelihood that their findings will be detected and applied. The traditional practice of reporting findings in a single scholarly article may be enhanced by adding mentions of the research findings in these alternative media and forums.

#### Importance of various types of knowledge use

The respondents were asked to rank various types of knowledge use. Note that the first four choices shown in Table 3 all represent instrumental use of knowledge--that is, applying the knowledge as intended and in some practical form. The fifth option was left open ended.

**Table 3:** Ranking importance across various types of knowledge use

	Very Important	Important	Moderately important	Of little importance	Unimportant	Not applicable
**To create or revise industry standards or clinical protocols is ...**	AHEAD ASHA OSERS RESNA	ATIA	ISAAC			

**To build laboratory instruments or clinical tools is ...**	RESNA	ASHA OSERS		ATIA	ISAAC	AHEAD

**To create freeware (hardware, software) for free download or access is ...**		OSERS	ISAAC	RESNA		ATIA AHEAD ASHA

**Designing new or improved commercial devices or services is ...**	ATIA RESNA	ISAAC ASHA OSERS				AHEAD

**For other purposes is ... -Promote the AT field-Inform policy or practice**		ATIA RESNA AHEAD				

ATIA = Assistive Technology Industry Association; AHEAD = Association on Higher Education and Disability; ISAAC = International Society for Augmentative and Alternative Communication; ASHA = American Speech-Language Hearing Association; OSERS = Office of Special Education and Rehabilitative Services; RESNA = Rehabilitation Engineering & Assistive Technology Society of North America; AT = assistive technology.

For the open-ended responses, three organizations reported as important the use of new research knowledge for conceptual--rather than instrumental--purposes. These organizations use new knowledge to promote a related idea that is consistent with the findings but of a more abstract nature, such as promoting the field of assistive technology, informing policy, or informing practice.

There are two issues associated with the documentation of research-result applications. On the one hand, the evaluation problem of demonstrating the utility of research results with evidence from applications requires a systematic effort to track those instances. Organizations such as the ones studied in this project are good sources of information about applications. This evidence is beneficial for the grantee and the sponsor alike, as it shows that someone beyond the knowledge creator deemed it worthy of attention. Furthermore, given new expectations, a new level of evidence is necessary for verifying the utility of the applied knowledge to the recipient audience. Demonstrating that the research-based knowledge was useful to the recipient requires establishing two-way communication, with feedback from the recipient. The participating national organizations are already doing so as part of their service to their constituencies, so they become an important source of evidence to show utility.

On the other hand, from the point of view of what it takes for KT to happen, these organizations reveal that dedication to the multiple forms of interface with constituencies is indispensable. The effort of translation seems to be comparable in scale to the research effort itself and could potentially be greater if one considers that for each line of research work, multiple potential uses could arise if knowledge flow is facilitated.

#### Feedback from target audiences

We explored the organization's procedures for securing feedback from their target audiences, problems they encountered when verifying the utility of information, and what solutions were applied. All but one organization described structured-feedback mechanisms:

• Member surveys (ATIA, AHEAD, ASHA)

• Special interest groups (ATIA, RESNA)

• Semi-structured feedback, such as listservs (ATIA, ASHA, RESNA)

• Formal reporting mechanisms for grantees (OSERS)

This constitutes further evidence regarding the complex nature of successful interaction with knowledge user communities to facilitate knowledge flow. Such mechanisms are intended to address that complexity, as well as to collect data for program evaluation purposes.

This complexity is echoed by the problems organization's reported when obtaining feedback, such as the diversity of people involved with these national organizations, the heterogeneity of their perspectives regarding new knowledge, and the inability to follow-up over time. These issues must be overcome in order to document how members apply the knowledge to generate outcomes and how those outcomes eventually impact the constituents the organizations hope to benefit. The KT solutions they already apply include ensuring that staff members are sensitive to the diverse range of stakeholders comprising membership and providing technical assistance to ensure knowledge users comprehend the material. It is apparent that enhancing effective communication of research knowledge to targeted audiences requires active planning and management rather than passive diffusion.

To complete their reporting on knowledge use, the national organizations each provided two examples of using research-based findings with either internal or external audiences. The examples showed how these organizations scour the journals and other sources of research output with the needs of their constituencies in mind. They then develop a mechanism to convey those research results in a manner that makes it accessible to the relevant audience. Most examples included a new media format or the choice of a specific diffusion channel accessible to the relevant constituencies. In one case, the research result led to the implementation of an active institutionalized mechanism of direct application in the constituency itself. This reinforces the main point that KT involves not only the knowledge being translated but also an understanding of the context in which the knowledge may be applied and the means for communicating within those specific contexts, all to achieve the objectives of knowledge use and its documentation. The research results are but one input for that broader process.

### 7. Incentives for seeking or applying research knowledge

Given that all organizations are engaged in various forms of knowledge generation, assessment, and application, they reported a variety of incentives to encourage their members and associates to search for or apply research knowledge. The questionnaire provided four defined categories of incentives and requested that they specify any others in a fifth open category.

All six organizations reported using workshops, webcasts, or preconference training. Four organizations use continuing education units or discounts for advanced conference registration. Only two use certificates of course/program completion. In the open category, one organization reported using strand advisors from affiliated organizations, while another also uses listservs among colleagues.

These organizations clearly leverage the value inherent in operating education and training forums for members and constituents. All provide opportunities to encourage awareness, interest, and use of research-based knowledge. Providing staff and members with discounts or special access is a low-cost yet high-return approach to encourage knowledge use.

Of particular note is the network of strand advisors from other national organizations that ATIA uses. These partners bring their own organization's particular expertise to conference and workshop agendas, peer review, and technical assistance. From a KT perspective, these strand advisors provide the knowledge creator with additional insights about potential target audiences and new collaborators for customizing the form and content of knowledge packages to their values and interests.

### 8. Measurement of awareness, interest, or application of new knowledge

The problem of measurement is difficult even as a research issue, so it is not surprising that there is no standard approach to measuring knowledge use among internal staff, members, or constituents across these organizations. However, in all but one of our cases a significant effort is devoted to in some way gauging one or more of these dimensions of knowledge use. The four organizations conducting annual conferences conduct post-session evaluations to track audience perceptions of content delivered. AHEAD reports no formal efforts to measure knowledge use. ISAAC and ASHA track the impact factor ratings for their peer-reviewed journal. OSERS relies on the apparent influence of new knowledge as observed in grant applications reviewed by internal staff. RESNA monitors requests for information on particular topics, particularly through listserv threads. The differences are obviously related to the different missions and constituencies of each organization, and many are related to generic interests in knowledge use rather than KT itself.

ASHA describes the most structured approach to measuring knowledge use. Every three years ASHA conducts a "Knowledge, Attitudes and Practices" survey, which includes questions about incorporating research-based evidence into practice. Over time, this approach could provide a rich set of information regarding trends in research and in practice. Overall, it appears that the field would benefit from an instrument capable of measuring awareness, interest, and use of research-based knowledge. The lead author is involved in such an effort to be reported in an upcoming publication.

### 9. Postgraduate training of organizations' staff

The training of an organization's staff will have a significant role in determining its ability to acquire new research-based knowledge. In the cases reported here, the percentage of staff with postgraduate training varies widely. Three report more than 80% of staff have postgraduate training, while three report that about half of their staff do. Two organizations do not track education, as their multisector constituents include higher percentages of entry-level professionals, manufacturers, and consumers. Knowing the educational level of the staff helps external researchers calibrate the level of sophistication inherent in the materials they prepare for presentation to these organizations. However, facilitating the flow of research knowledge to various constituencies requires both comprehension of the findings and representation of the audience's capacity to understand them, so a range of education and experience may help with effective translation.

### 10. Recommendations for improving communication with researchers

Respondents considered ways for improving the flow of research knowledge to and through them. All six suggested opportunities for increased engagement with them by individual researchers. All mentioned the need to have someone take the time to "translate" research findings from the academic language of the scholarly article to the practical language of the clinician, consumer, or manufacturer. Given the reluctance to independently translate research knowledge reported in Section 3, it is not surprising that organizations seek assistance from the research community, if not from the original study authors themselves. They want someone to explain the findings, explain their implications for the audience, and suggest ways to implement the findings within an action framework. Three requirements for this are succinctly summarized in the evaluation literature as, "What? So What? Now What?" [13].

Expectations for this translation task include using language appropriate to the audience, summarizing the findings in the context of a case example, preparing "distribution-ready" materials in user-friendly formats, and preparing multiple versions of the findings for communication through less formal media such as websites, newsletters, and email lists. Respondents speak of making the knowledge more "digestible" for their targeted members or constituents. This is partly a matter of effective communication but also a matter of convenience. To the extent a researcher delivers materials already tailored to a particular audience, the national organization can efficiently process that material for delivery to the audience at little cost in terms of time or resources.

ATIA spoke specifically about opportunities to establish closer links between researchers and manufacturers. These links may identify opportunities to integrate findings into product development activities, to have researchers generate knowledge needed by a corporation, or to integrate research findings into product launch or marketing materials. All suggestions demonstrate an awareness of the value of research to their memberships.

OSERS suggested formalizing the communication and translation portion of interactions, including all of the different stages and steps involved. At a minimum, an easy-to-follow framework for application at the level of middle management could help organizations facilitate the flow of knowledge between the creators and the user audiences.

## Conclusions

Government sponsors and their grantees are challenged to demonstrate that their project outputs are reaching stakeholders and to generate evidence of knowledge uptake and use by these stakeholders. They may benefit from strategies for reaching the targeted audiences and then collecting evidence of use. This study explored the potential for national organizations to participate in the translation and dissemination of knowledge.

The main finding is good news. All of the national organizations studied--due to their link to nonacademic stakeholder groups--demonstrate attributes that are critical mediators in the knowledge flow from research outputs to awareness interests, application, and realization of potential benefits. However, the manner in which this mediation occurs is different for each national organization. In this study, the selected national organizations involved with the field of AAC value research-based knowledge in very specific ways linked to the interests of their constituencies. Most are involved in either creating knowledge or identifying knowledge created by others. Even though they all acquire research knowledge in some way (*e.g*., performing research, providing summaries, or conversions to other media), they all recognize the challenge of interpretation of results in ways relevant to their constituencies while preserving the validity and quality of the original research.

In the process of identifying useful results from research, all the cases showed that a significant effort was made to engage with the knowledge creators themselves. This feedback path enhances the translation potential of organizations, which varies across the organizations. Those with internal research capabilities have more fluid interaction with researchers due to their shared understanding of the academic context of research. Organizations that are more consumer or end-user oriented require other formats of interaction and showed different ways to engage with research results.

The principles of KT suggest that the researchers have a responsibility complementary to an organization's ability to acquire new knowledge [4,14], that is, to tailor their findings to the capacities and values of the target audiences to make the knowledge more absorbable to nonscholars. Researchers who tailor the format, content, and context of their findings in such a manner should be rewarded with higher levels of absorption. This, in turn, should result in higher levels of awareness, interest, and, possibly, use.

Another critical role for these organizations is the creation of forums and social loci where interested parties focused on an area of research results can interact to develop an agenda for future translation. Many of the communication mechanisms applied with their constituencies produce up-to-date information about the context of use. These could be studied across organizations as a natural follow-up to this KVM exercise.

These organizations routinely facilitate the flow of knowledge in ways consistent with the various models of KT, as summarized in Sudsawad [14]. For example, they are engaged in many of the activities described in the KTA framework, such as knowledge creation and synthesizing and tailoring knowledge outputs, both within the knowledge-creation component [4]. All the organizations engage in identifying, reviewing, and selecting knowledge that constitutes the stage of the KTA cycle that connects the knowledge creation with the action sides of the process. They also adapt knowledge to local contexts of users, assessing barriers to use and sustaining knowledge use. However, none of the organizations report a substantial role in interventions, monitoring, and evaluation of knowledge use. It seems to be in the interest of sponsors and researchers to engage them to expand their roles into these evidence-rich areas of outcomes and impacts.

National organizations are involved in KT activities spanning the research context, various user contexts, and their own intermediary context. Therefore, it is of fundamental importance to identify and understand the critical interfaces for knowledge flow in each area of research that has potential for expanded use in society. Organizations and members valuing research-based knowledge can strive to optimize their ability to acquire knowledge on the state of the sciences. Conversely, knowledge creators interested in increased use can maximize the absorbability of the knowledge to facilitate translation to target audiences. The combination will ensure the highest possible return on public investment in research activities.

This KVM study concludes that national organizations are good sources of evidence of actual and potential use. It behooves sponsors and researchers to engage such organizations to meet statutory requirements and general expectations for increased use of research-based knowledge.

## Competing interests

The authors declare that they have no competing interests.

## Authors' contributions

JPL designed the study, led the case study interview team, drafted the questionnaire, performed the initial data analysis, and prepared the initial manuscript. JDR pioneered the KVM concept and guided implementation in this context, revised the questionnaire, conducted a secondary analysis, and contributed substantive revisions throughout the manuscript. Both authors have read and approved the final manuscript.

## Supplementary Material

Additional file 1 **appendix A**. Knowledge value mapping in national organizations. Click here for file
