# Supplementary material for: Engaging national organizations for knowledge translation: Comparative case studies in knowledge value mapping
Source: Implement Sci. 2011 Sep 12;6:106. doi: 10.1186/1748-5908-6-106 (PMC3180429; doi:10.1186/1748-5908-6-106)
Supplement: Additional file 1 — appendix A. Knowledge value mapping in national organizations. [file 1748-5908-6-106-S1.DOC]

**Additional file 1, appendix a. Knowledge value mapping in national organizations**

**Knowledge value mapping in national organizations**

**How does your organization interact with knowledge generated through research studies?**

This survey explores how your organization interacts with knowledge generated by research studies. We are profiling multiple national organizations to help researchers understand how to communicate their research findings to people with related interests, and thereby increase the rate at which new knowledge generated through their research studies is used by others. The study is focused on organizations and members interested in rehabilitation and assistive technology devices and services that benefit persons with disabilities.

Name:

Title:

Organization:

NOTE: The survey explores six ways in which your organization may interact with knowledge from research:

1) **Creating Knowledge:** Conducting research internally or funding others to do research for your organization;

**2) Identifying Knowledge:** searching for research findings that have already been produced by others;

**3) Translating Knowledge:** paraphrasing research findings to make them more relevant and understandable;

**4) Adapting Knowledge:** interpreting research findings to improve their fit within your organization’s context;

**5) Communicating Knowledge:** disseminating or demonstrating research findings through various media;

**6) Using Knowledge:** applying research findings to situations within your organization or membership;

Please answer the following questions to the best of your ability, speaking on behalf of your organization. There will be an opportunity to have questions clarified during our follow-up telephone call.

**Question #1.** Relative to other activities, how frequently does your organization engage in **Creating Knowledge** through Research activity? That is, conduct or perform your own research or pay/fund others to do research for you? Choose the answer which most closely fits your organization.

[ ] Very Frequently

[ ] Frequently

[ ] Occasionally

[ ] Rarely

[ ] Very Rarely

[ ] Not Applicable – **SKIP to QUESTION #2**

1a) For what purpose are you conducting research or funding research performed by others?

1b) Who conducts the research?

[ ] An internal department or staff member

[ ] An outside service contractor/grantee

[ ] Both an internal department or staff member and an outside contractor/grantee

1c) Who are the main intended users of the research knowledge your organization creates? Please check all that apply.

[ ] Our internal organizational staff

Organizational members who are:

[ ] Clinician/Practitioners

[ ] Consumer/Family

[ ] Policy Makers

[ ] Manufacturer/Suppliers

[ ] Educators/Employers

[ ] Non-members who are (describe below):

**Question #2.** Relative to other activities, how frequently does your organization engage in **Identifying Knowledge** from Research activity? That is, searching for research findings that have already been produced by others? Choose the answer which you think most closely fits your organization.

[ ] Very Frequently

[ ] Frequently

[ ] Occasionally

[ ] Rarely

[ ] Very Rarely

[ ] Not Applicable – **SKIP to QUESTION #3**

2a) For what purpose or reason is your organization looking for research findings produced by others?

2b) What sources does your organization search when identifying new research knowledge? Please check all that apply.

[ ] Academic Journals (online or print)

[ ] White papers or other in-house reports from other organizations

[ ] Newspapers or Magazines

[ ] Websites

[ ] Trainings or Conference

[ ] Individual Experts

[ ] Other (describe below):

2c) For each source checked in question 2b, please provide names of the specific sources your organization finds credible for providing new research knowledge.

Academic Journals:

Newspapers or Magazines:

Websites:

Trainings or Conferences:

Other:

2d) Please describe any policies or procedures your organization uses to judge the quality of the research findings?

**Question #3**. Relative to other activities, how frequently does your organization engage in **Translating Knowledge** from Research activity? That is, paraphrasing research findings to make them more relevant and understandable to your organization and members? Choose the answer which you think most closely fits your organization.

[ ] Very Frequently

[ ] Frequently

[ ] Occasionally

[ ] Rarely

[ ] Very Rarely

[ ] Not Applicable – **SKIP to QUESTION #4**

3a) To what extent does your organization typically paraphrase research findings?

3b) Describe the qualifications (training, certificates, experience or education) of the people who are responsible for translating the research findings?

3c) Who does your organization translate research for? Check all that apply.

[ ] Our internal organizational staff

Organizational members who are:

[ ] Clinician/Practitioners

[ ] Consumer/Family

[ ] Policy Makers

[ ] Manufacturer/Suppliers

[ ] Educators/Employers

[ ] Non-members who are (describe below):

3d) Please provide an example of complications that have arisen when your organization translated or paraphrased research findings, and explain how you overcome them?

**Question #4**. Relative to other activities, how frequently does your organization engage in **Adapting Knowledge** from research activity? That is, interpreting research findings to improve their fit within your organization’s context? Choose the answer which you think most closely fits your organization.

[ ] Very Frequently

[ ] Frequently

[ ] Occasionally

[ ] Rarely

[ ] Very Rarely

[ ] Not Applicable – **SKIP to QUESTION #5**

4a) Please provide some examples of how your organization has interpreted knowledge from research findings to make it fit within the context of your own program or issue?

4b) Why were these adaptations to the existing research findings necessary?

**Question #5.** Relative to other activities, how frequently does your organization engage in **Communicating Knowledge** from research activities? That is, disseminating or demonstrating research findings through various media? Choose the answer which you think most closely fits your organization.

[ ] Very Frequently

[ ] Frequently

[ ] Occasionally

[ ] Rarely

[ ] Very Rarely

[ ] Not Applicable – **SKIP to QUESTION #6**

5a) What criteria does your organization use to decide which research knowledge is communicated and to whom it is communicated?

5b) What formats does your organization use to distribute research knowledge? Please check all that apply.

[ ] E-mail/Listserv

[ ] Website

[ ] Periodicals (Journals, Magazines, Newsletter…)

[ ] White papers or other in-house reports

[ ] Conference Proceedings

[ ] Conference Presentations/Workshops

[ ] Webcasts/Webinars

[ ] Special Interest Group Interactions

[ ] Other:

5c) Who are the target audiences for your organization’s knowledge communication efforts?

[ ] Our internal organizational staff

Organizational members who are:

[ ] Clinician/Practitioners

[ ] Consumer/Family

[ ] Policy Makers

[ ] Manufacturer/Suppliers

[ ] Educators/Employers

[ ] Non-members who are (describe below):

5d) Please describe any complications or problems that have arisen when your organization has communicated research findings and how you overcame them?

**Question #6.** Relative to other activities, how frequently does your organization engage in **Using Knowledge** from research activities? That is, apply research findings to situations within your organization or membership? Choose the answer which you think most closely fits your organization.

[ ] Very Frequently

[ ] Frequently

[ ] Occasionally

[ ] Rarely

[ ] Very Rarely

[ ] Not Applicable – **SKIP to QUESTION #7**

6a) Please provide some general examples of how your organization has directly used knowledge from research findings?

6b) What were the sources of that new knowledge? Please check all that apply.

[ ] Your Organizations own Research (or commissioned/funded research)

[ ] Academic Journals (Online or not)

[ ] White papers or similar reports from other organization reports

[ ] Newspapers or Magazines

[ ] Websites

[ ] Trainings or Conference

[ ] Other (describe below)

6c) Please rate the following five statements in terms of their importance to your organization. Circle or highlight the appropriate importance level 1-5, where 5 = very important and 1 = unimportant.

|  | Very Important | Important | Moderately Important | | Of Little Importance | Unimportant | Not Applicable |  |
| --- | --- | --- | --- | --- | --- | --- | --- | --- |
| a. For my organization, Using Knowledge to create or revise Industry Standards or Clinical Protocols is… | 5 | 4 | 3 | | 2 | 1 | N/A |  |
| b. For my organization, Using Knowledge to produce Laboratory Instruments or Clinical Tools is… | 5 | 4 | 3 | | 2 | 1 | N/A |  |
| c. For my organization, Using Knowledge to develop Freeware such as software to download or instructions for building hardware is… | 5 | 4 | 3 | 2 | | 1 | N/A | |
| d. For my organization, Using Knowledge to produce New or improved Commercial Devices or Services that will be available in the marketplace is… | 5 | 4 | 3 | 2 | | 1 | N/A | |
| e. For my organization, using Knowledge for other purposes [described below] is...  Please explain: | 5 | 4 | 3 | 2 | | 1 | N/A | |

6d) Does your organization have a procedure (for example, formal feedback from members; and informal observation of member interactions) for verifying the usefulness of new knowledge? If so, please describe.

6e) Please describe any problems or complications when trying to verify the usefulness of new knowledge.

6f) What information or resources helped your organization overcome these problems?

6g. Please provide one or two specific examples of your organization’s use of a new research finding. For each example, include: (i) a detailed description of what the research finding was and how your organization used it; (ii) the source of the research finding and how it was found; (iii) specific groups or individuals who used the new knowledge (internally or externally):

Example 1

(i) Description of finding and how it was used?

(ii) Source of research finding and how found?

(iii) Specific Users of the knowledge:

Example 2

(i) Description of finding and how it was used?

(ii) Source of research finding and how found?

(iii) Specific Users of the knowledge:

**Question #7.** Please describe any incentives that your organization uses to encourage your internal associates or members to become aware of, or apply new research-based knowledge. Check all that apply.

[ ] Continuing Education Units (CEUs)

[ ] Certification of Completion/Attendance

[ ] Discount on advanced conference registration

[ ] Offering Workshops, Webcasts or Pre-Conference Training

[ ] Other, please describe:

**Question #8.** How does your organization measure the levels of awareness, interest or application of new knowledge among your memberships? What is being measured in each case? (For example, post workshop surveys to measure awareness and interest, post conference follow up surveys to measure application)?

**Question #9.** What percentage of your members have education/training in a research field equivalent to a Masters or Doctoral degree?

__________%

**Question #10.**  Can you identify or suggest any ways in which researchers could help your organization facilitate the flow of knowledge from them as the sources, through your organization and out to your members?

Thank you for spending some of your valuable time with our questionnaire. We look forward to receiving your responses by email and to our follow up call to answer any questions and to discuss your organization’s engagement with research-based knowledge.
